# Radioligand therapy in the therapeutic strategy for patients with gastro-entero-pancreatic neuroendocrine tumors: a consensus statement from the Italian Association for Neuroendocrine Tumors (Itanet), Italian Association of Nuclear Medicine (AIMN), Italian Society of Endocrinology (SIE), Italian Association of Medical Oncology (AIOM).

**DOI:** 10.1007/s40618-024-02448-6

**Published:** 2024-10-12

**Authors:** Francesco Panzuto, Manuela Albertelli, Maria Luisa De Rimini, Francesca Maria Rizzo, Chiara Maria Grana, Mauro Cives, Antongiulio Faggiano, Annibale Versari, Salvatore Tafuto, Nicola Fazio, Annamaria Colao, Federica Scalorbi, Diego Ferone, Saverio Cinieri, Marco Maccauro

**Affiliations:** 1 Department of Medical-Surgical Sciences and Translational Medicine, Digestive Disease Unit, Sapienza University of Rome, Sant’Andrea University Hospital, ENETS Center of Excellence, Rome, Italy; 2 Endocrinology, Department of Internal Medicine and Medical Specialties (DiMI), University of Genova, Genova, Italy; 3 Endocrinology, IRCCS Ospedale Policlinico San Martino, Genova, Italy; 4 Nuclear Medicine - PET and Therapy Unit, Dept. of Health Services AORN dei Colli, Naples, Italy; 5 Oncology and Breast Unit, Ospedale “A. Perrino”, Brindisi, Italy; 6 Radiometabolic Therapy, Nuclear Medicine Division, IRCCS IEO European Institute of Oncology, Milano, Italy; 7 Interdisciplinary Department of Medicine, University of Bari “Aldo Moro”, Bari, Italy; 8 Unit of Endocrinology, Department of Clinical and Molecular Medicine, ENETS Center of Excellence, Sant’Andrea Hospital, Sapienza University, Via di Grottarossa 1038, Rome, 00189 Italy; 9 Department of Nuclear Medicine, Azienda Unità Sanitaria Locale-IRCCS of Reggio Emilia, Reggio Emilia, Italy; 10 Sarcomas and Rare Tumors Unit, Istituto Nazionale Tumori, IRCCS Fondazione “G. Pascale”, Naples, 80131 Italy; 11 Division of Gastrointestinal and Neuroendocrine Tumors Medical Treatment IEO, European Institute of Oncology IRCCS, Milan, Italy; 12 Unit of Endocrinology, Diabetology and Andrology, Department of Clinical Medicine and Surgery, University of Naples Federico II, Naples, Italy; 13 UNESCO Chair “Education for Health and Sustainable Development”, University of Naples Federico II, Naples, Italy; 14 Nuclear Medicine Department, Foundation IRCCS, Istituto Nazionale Tumori, Milan, Italy

**Keywords:** Peptide-receptor radionuclide therapy, Radioligand therapy, Neuroendocrine tumors, Somatostatin analogs, Chemotherapy, Everolimus, Sunitinib, Guidelines

## Abstract

**Purpose:**

This paper outlines the consensus of the Italian Association for Neuroendocrine Tumors(Itanet), the Italian Association of Nuclear Medicine (AIMN), the Italian Society of Endocrinology (SIE), and the Italian Association of Medical Oncology (AIOM) on treating neuroendocrine neoplasms (NENs)with radioligand therapy (RLT).

**Methods:**

A list of 10 questions regarding using RLT ingastroenteropancreatic neuroendocrine tumors (GEP-NETs) was addressed after a careful review of theavailable literature. compiling information from the MEDLINE database, augmented with expert opinionsand recommendations, aligns with the latest scientific research and the author’s extensive knowledge.The recommendations are evaluated using the GRADE system, showcasing the level of evidence andthe strength of the recommendations.

**Results and Conclusions:**

Specifically, this paper focuses on thesubcategories of well-differentiated gastroenteropancreatic neuroendocrine tumors (GEP-NETs) thatexpress somatostatin receptors and are considered suitable for RLT, according to internationalguidelines.

**Supplementary Information:**

The online version contains supplementary material available at 10.1007/s40618-024-02448-6.

## Introduction

Neuroendocrine neoplasms (NENs) comprise a heterogeneous group of malignancies arising from the diffuse neuroendocrine cell system. Gastroenteropancreatic (GEP) NENs represent the most common subtype, with an increasing worldwide incidence over the past decades [1]. According to their histopathological features, mitotic count, and Ki-67 index, GEP-NENs are classified as neuroendocrine tumors (NETs) or neuroendocrine carcinomas (NECs). GEP-NETs are well-differentiated neoplasms, defined as grade G1 (Ki-67 < 3%, mitotic count < 2/2 mm²), G2 (Ki-67 3–20%, mitotic count 2–20/2 mm²), or G3 (Ki-67 > 20%, mitotic count > 20/2 mm²). In contrast, GEP-NECs are aggressive and poorly differentiated neoplasms G3 (Ki-67 > 20%, mitotic count > 20/2 mm²) [2]. The majority of GEP-NETs are sporadic and non-functional [3]. Therapy goals encompass tumor excision with curative intent and/or the halting of disease progression, and the control of clinical symptoms in functional NETs. Surgery, if feasible, represents the primary and only curative approach for localized GEP-NET G1 or G2 but may also be considered in the context of advanced NETs for palliative resection, debulking surgery, or hepatic metastasectomy. At diagnosis, up to 80% of GEP-NETs are locally advanced or metastatic; therefore, non-surgical strategies such as somatostatin analogs (SSA), radioligand therapy (RLT), targeted therapies with the mTOR inhibitor everolimus or the multiple tyrosine kinase inhibitor sunitinib, and systemic chemotherapy, should be evaluated. Specifically, RLT is an effective and relatively safe option that has been investigated for over 20 years in well-differentiated NETs expressing somatostatin receptors (SSTR). RLT involves of administering radionuclide-labeled SSA, which selectively targets NET cells. The role of RLT in NENs is evolving, and novel strategies are under evaluation, including the implementation of new radiopharmaceuticals, combination with other therapies, or intra-arterial administration [4]. Currently, [177Lu]Lu-[DOTA0,Tyr3]-octreotate (177Lu-DOTATATE) is indicated for unresectable, metastatic or locally advanced, G1 or G2, SSTR-positive GEP-NETs as a second-line option after SSA. The approval by the European Medicines Agency (EMA) in 2017 and the US Food and Drug Administration (FDA) in 2018 was strongly encouraged by the hallmark phase III NETTER-1 trial [5], which demonstrated a significant improvement in PFS, response rate, and quality of life (QoL) in the 177Lu-DOTATATE arm compared to high-dose octreotide (60 mg/month) in patients with advanced midgut NETs progressive on SSA. To date, the optimal therapeutic algorithm for GEP-NETs, comprising the role of RLT, has not been standardized. Current clinical practice considers RLT when progression occurs on previous pharmacological treatment. The European Neuroendocrine Tumor Society (ENETS) supports the role of RLT in intestinal NETs as second-line therapy after the failure of SSA or as third-line therapy after the failure of everolimus [6]. Regarding pancreatic NETs (panNETs), RLT is recommended in lower-grade NETs in case of progression after SSA, chemotherapy, or targeted drugs (everolimus/sunitinib) [7]. The European Society of Medical Oncology (ESMO) guidelines encourage considering RLT earlier in the treatment sequence, especially in panNETs. According to ESMO guidelines, RLT is recommended as second-line therapy in progressive midgut NETs after SSA but may also be considered in carefully selected NET G3 cases [8]. Both ENETS and ESMO guidelines recognize the role of RLT in managing carcinoid syndrome or functional NETs refractory to SSA [8, 9]. Given the non-complete uniformity of the current recommendations, it is crucial to provide clinicians with clear and well-structured guidance for personalized therapeutic decisions in real-world clinical practice. Therapy should be tailored to each patient according to tumor pathological and functional status, SSTR imaging, patient choice, and comorbidities. Therefore, multidisciplinary care of patients affected by GEP-NETs at referral centers is pivotal in integrating and optimizing diagnostic and therapeutic strategies [10].

## Methods

This work was developed by representatives from each of the participating scientific societies. After an initial web meeting, 10 questions were identified, focusing on the role of RLT in GEP-NETs, as detailed in Table 1. The questions were limited to sporadic, well-differentiated tumors, excluding high-grade NEC and non-sporadic tumors related to hereditary syndromes. Hence, the manuscript consistently uses the term “NET” in this context. Each question was addressed by a specialized team from the societies, leveraging their expertise. They conducted a PubMed literature search using the following keywords: (“radioligand therapy” OR “peptide receptor radionuclide therapy” OR “PRRT”) AND (“gastroenteropancreatic neuroendocrine tumors” OR “GEP-NETs” OR “gastroenteropancreatic NETs” OR “gastrointestinal neuroendocrine tumors” OR “pancreatic neuroendocrine tumors”). Since 177Lu-Dotatate is the only therapy approved by authorities for treating patients with GEP-NET, the literature search was limited to articles covering 177Lu-DOTATATE exclusively. Studies focusing on treatments with other radioligands were considered outside the scope of this work. Recommendations are provided based on the highest quality evidence available and the collective expertise of the authors. These are categorized by both the level of evidence (ranging from 1 to 5) and the strength of the recommendation (graded A to D), as outlined in suppl. Table according to the GRADE system [11].

**Table 1:** List of questions

1. Who is the potential candidate for treatment with RLT?
2. How should progressive disease be defined before planning RLT?
3. If and how does the FDG PET influence the decision to perform RLT?
4. What is the evidence for choosing RLT versus targeted agents after the failure of somatostatin analogues?
5. What is the evidence for choosing RLT versus chemotherapy after the failure of somatostatin analogues?
6. What is the evidence for choosing RLT versus high-dose SSA after the failure of standard dose SSA in NF NETs?
7. How and when should the efficacy of RLT be monitored after initiating treatment?
8. How to manage frail patients who are to undergo RLT?
9. Is there a room for RLT in G3 GEP-NETs?
10. Is there a rationale for repeating RLT treatment?

The manuscript was refined through textual email discussions and virtual meetings in October 2023, January 2024, and April 2024, leading to a consensus draft. After external review and approval from the executive boards of all societies, the final draft was endorsed.

### Statements

#### Q1. Who is the potential candidate for treatment with RLT?

RLT with 177Lu-DOTATATE is currently approved by both the Food and Drug Administration (FDA) and the European Medicines Agency (EMA) for the treatment of unresectable or metastatic, progressive, well-differentiated, G1/G2, SSTR-positive GEP-NETs. This indication is based on the multicenter, phase III, randomized, open-label NETTER-1 trial [5] and large retrospective cohort studies [12, 13]. The NETTER-1 trial [5] randomized 229 patients with well-differentiated, metastatic midgut NETs who progressed on standard dose octreotide LAR to receive either 177Lu-DOTATATE at 7.4 GBq every 8 weeks or octreotide i.m. at 60 mg every 4 weeks. The estimated rate of PFS at month 20 was 65% in the 177Lu-DOTATATE arm and 11% in the control arm (HR: 0.21, *P* < 0.0001), with consistent benefits across major prespecified subgroups. Moreover, RLT with 177Lu-DOTATATE significantly improved many QoL domains compared with high-dose octreotide [14]. While the NETTER-1 trial enrolled only patients with midgut NETs, a large body of evidence suggests that RLT with 177Lu-DOTATATE is also safe and effective in SSTR-positive pancreatic and hindgut primaries [12, 13, 15]. More recently, the multicenter, phase III, randomized, open-label NETTER-2 trial has investigated 177Lu-DOTATATE plus octreotide versus high-dose octreotide in patients with newly diagnosed, advanced, SSTR-positive G2/G3 GEP-NETs with Ki-67 ranging between 10% and 55% [16]. The median PFS was significantly prolonged in the investigational arm (22.8 months) compared to the control arm (8.5 months; stratified HR: 0.28, *p* < 0.0001), with a significantly higher overall response rate (ORR) in the 177Lu-DOTATATE arm (43%) versus the high-dose octreotide arm (9.3%; OR: 7.81, *p* < 0.0001). On this basis, likely, regulatory authorities will formally expand the indications for RLT to include frontline treatment of patients with GEP-NETs harboring a Ki-67 between 10% and 55%.

At present, potential candidates for RLT with 177Lu-DOTATATE include patients with advanced SSTR-positive GEP-NETs who have progressed on prior SSA therapy. Since high tumor burden negatively impacts the efficacy of RLT [15], early placement of RLT in the therapeutic algorithm is advocated. Therefore, all patients with SSTR-positive advanced GEP-NETs progressive on first-line treatment should be considered for RLT. In patients with bulky, symptomatic disease (particularly in the case of pancreatic primaries) who need rapid tumor shrinkage, chemotherapy might be preferred over RLT. In the future, potential candidates for RLT will also include patients with newly diagnosed G2/G3 GEP-NETs and Ki-67 ranging between 10% and 55%. The progressive expansion of the patient population potentially amenable to treatment with 177Lu-DOTATATE, in line with the advent of 177Lu-PSMA-617 for the treatment of prostate cancer [17], might pose several challenges from a production and drug administration standpoint. Timely preparation is needed to avoid bottlenecks and allow the administration of RLT to all potential candidates without delays.

#### Recommendation

The candidate for RLT is a patient with advanced (unresectable or metastatic) SSTR-positive GEP-NET who has progressed on prior therapy with SSA. For these patients, early incorporation of 177Lu-DOTATATE RLT into the treatment algorithm is recommended (1b - A).

#### Q2. How should progressive disease be defined before planning RLT?

Assessing disease progression in GEP-NETs before planning RLT involves a thorough evaluation using various clinical, imaging, and laboratory methods. Here are the key steps and considerations in assessing disease progression.

Imaging Studies: Utilize radiological imaging such as computed tomography (CT) and magnetic resonance imaging (MRI) scans to assess evidence of primary tumors and metastasis and estimate tumor burden [18]. These investigations help quantify neoplastic infiltration, pleural or ascitic fluid volume, and the presence of carcinoid heart disease (evaluated by echocardiography). CT and MRI also identify previously unrecognized lesions or conditions needing urgent treatment, such as pathological spinal fractures, and are essential for ruling out indications for locoregional therapies like embolization or chemoembolization in patients with liver-only disease [19].

Functional Imaging: Functional imaging, particularly 68-Gallium-SSTR PET scans (SSTR-PET), is specific for NETs [18]. This imaging modality helps identify the presence of SSTRs on tumor cells, guiding the selection of patients suitable for RLT. For lesions with high proliferative indexes, [18 F]FDG PET/CT may complement the assessment by visualizing heightened metabolic activity, thus refining the evaluation of lesions targeted with alternative therapies [20, 21]. Recent advancements include the introduction of volumetric parameters like SSR-derived tumor volume and total lesion SSR as tools to aid in predicting PFS before RLT [22].

Biomarkers: While specific tumor markers are assessed in functioning tumors associated with clinical syndromes, the use of biochemical markers like chromogranin A, alkaline phosphatase, or alterations in transaminase ratios, has been proposed to predict therapy effectiveness, although without definitive evidence of their predictive significance [23–25]. Elevated chromogranin A levels alone should not be considered definitive evidence of disease progression due to the marker’s low specificity.

Histological Evaluation: For long-term survivors with multiple secondary disease localizations and historical biopsies, it’s crucial to consider a further histological evaluation before planning RLT due to the potential change in tumor grade over time [26]. This is especially pertinent if the historical biopsy was from the primary tumor and there has been a significant increase in metastatic lesion number and sites. Performing an [18 F]FDG PET/CT scan may help guide the selection of the most aggressive metastasis for biopsy.

Clinical Symptoms: Assess the patient’s symptoms, including changes in flushing, diarrhea, abdominal pain, or other related symptoms. Worsening or new symptoms may indicate disease progression, necessitating a CT, MRI, or PET scan to provide a comprehensive overview of the patient’s clinical condition.

Multidisciplinary Team Consultation: Engage a multidisciplinary team experienced in managing GEP-NETs, including oncologists, endocrinologists, gastroenterologists, radiologists, nuclear medicine specialists, pathologists, and surgeons, in the assessment process. Discuss the patient’s case to ensure a comprehensive understanding of the disease status and align with the patient’s will and expectations. Multidisciplinary management significantly enhances care levels in patients with GEP-NETs [27, 28].

It is essential to approach disease progression assessment in GEP-NETs using these methods. Treatment decisions are often based on a comprehensive evaluation of all available information, with plans typically personalized to each patient’s specific situation, considering factors like tumor grade, location, and overall health status.

#### Recommendation

An accurate multidisciplinary assessment of patients who are candidates for RLT is mandatory before initiating treatment. This assessment should include a complete radiological evaluation using CT and/or MRI, as well as SSTR-PET. In selected patients with a significant change in disease behavior—such as a noticeable increase in tumor lesions or an evident increase in tumor burden—performing [18 F]FDG PET/CT and/or repeating the histological evaluation may be proposed (3a - A).

#### Q3. If and how does the FDG PET influence the decision to perform RLT?

While [18 F]FDG PET/CT is not typically the primary imaging modality for GEP-NETs, it can be informative in certain cases and may influence decisions regarding RLT administration. EANM [29] and ENETS [30] guidelines recommend including [18 F]FDG PET/CT in the diagnostic pathway for higher G2 (Ki67: 10–20%), G3 NET, and NEC. The 2020 ESMO guidelines offer broader recommendations, suggesting the evaluation of both [18 F]FDG PET/CT and SSTR-PET for all G2-G3 NETs [8]. However, [18 F]FDG PET/CT can also be positive in low-grade NETs of the G1 type, maintaining an unfavorable prognostic significance even in these tumors, confirming that the role of this technique in low-proliferation forms still needs full clarification [31]. Some previous studies have investigated the use of both tracers, but they rely on retrospective data from populations that are not homogeneous regarding the primary lesion [32, 33]. SSTR-PET and [18 F]FDG PET/CT together may be indicated for certain cases, including at initial diagnosis for intermediate proliferative activity tumors and during follow-up when assessing treatment changes or discrepancies between radiological and clinical evaluations [34].

Here’s how [18 F]FDG PET/CT might influence the decision to perform RLT.

Tumor Metabolic Activity: [18 F]FDG PET/CT provides information about the metabolic activity of tumors. NETs are generally slow-growing and may not exhibit high glucose metabolism, making [18 F]FDG PET/CT less sensitive for these tumors. However, in poorly differentiated or more aggressive lesions with higher metabolic activity, [18 F]FDG PET/CT may be used to assess aggressive lesions’ presence, number, and location, guiding treatment decisions towards alternatives to RLT, such as chemotherapy [35, 36].

Tumor Intra and Inter-lesion Heterogeneity: GEP-NETs may exhibit heterogeneity in receptor expression and metabolic activity. Combining information from both radiotracers provides a more comprehensive view of tumor characteristics. For instance, elevated [18 F]FDG PET/CT activity might indicate swift progression in pancreatic NETs, even when early diagnosed or confirmed as well-differentiated. The presence of [18 F]FDG PET/CT uptake could indicate undifferentiated disease foci, significantly impacting therapy response and prognosis [37]. Lesions showing matched SSTR imaging with SSTR-PET and [18 F]FDG PET/CT uptake may suggest a good response probability to RLT, even in combination with chemotherapy [38].

Disease staging, monitoring, and therapeutic decision-making: the decision to perform RLT is based on the presence of SSTRs on tumor cells. If GEP-NETs show SSTR expression, RLT may be considered. However, in cases of uncertain diagnostic presentations (such as non-conclusive findings in CT, MRI, or SSTR-PET) or rapid clinical progression, it is advisable to also perform [18 F]FDG PET/CT for a comprehensive overview of the multi-metastatic disease.

Ultimately, the decision to perform RLT is multifaceted and should be made in consultation with a multidisciplinary team of specialists, considering the specific characteristics of the patient’s tumors and their responses to various imaging modalities and previous therapies. The goal is to tailor the treatment plan to the individual patient’s needs and the characteristics of their neuroendocrine lesions.

#### Recommendation

[18 F]FDG PET/CT is recommended before RLT in cases with heterogeneous uptake at SSTR-PET, and in patients with suspicion of rapidly progressive disease (3b - A).

#### Q4. What is the evidence for choosing RLT versus targeted agents after the failure of somatostatin analogues?

The phase 3 trials conducted on patients with intestinal NET reported that median PFS was not reached for RLT with 177Lu-Dotatate, while it was 11 months and 16.4 months for everolimus in non-functioning and functioning tumors, respectively [5, 39, 40]. Although these studies were designed on populations that are not directly comparable, the higher anti-proliferative efficacy of RLT compared with everolimus is now well established. This constitutes the first and most significant evidence in favor of choosing RLT after the failure of SSA treatment. The ORR was significantly higher with RLT than with everolimus [14, 39, 40]. In patients with advanced panNET initially considered unresectable or borderline, neoadjuvant treatment with 177Lu-Dotatate enabled successful surgery in 31% of cases [41]. Therefore, early use of RLT can alter these tumors’ natural history.

Patients with GEP-NET who are candidates to receive SSA as first-line therapy typically present with low-proliferating tumors and a long life expectancy. In this setting, the second-line therapy needs to be effective, but safety is of primary importance to avoid serious adverse events and related treatment interruptions or withdrawals. The ultimate goal is to achieve long-term tumor stabilization and a good QoL. For this purpose, RLT offers a better risk/benefit ratio than targeted therapies. By comparing different therapeutic sequences, RLT was found to be safer than either everolimus or chemotherapy as a second-line therapy [42, 43]. From the patient’s perspective, a French national survey indicated that RLT had the best median perceived tolerance compared to all other treatments, including everolimus, sunitinib, and chemotherapy [44]. On the other hand, toxicity, rather than tumor progression, was the most frequent reason for discontinuation of everolimus and sunitinib [44]. The long-term safety results of the NETTER-1 trial confirmed that 177Lu-Dotatate is safe, and no new serious adverse events were reported during the long-term follow-up [45].

Beyond the low toxicity rate, RLT has been reported to significantly impact health-related quality of life in large randomized trials performed in gastroenteropancreatic NETs, improving both global health status and specific symptoms [14, 46].

The phase II non-comparative OCLURANDOM study recently randomized patients with advanced, progressive, SSTR-positive panNET to receive either 177Lu-DOTATATE or sunitinib. The 12-month PFS rate was 80.5% in the RLT arm versus 42% in the sunitinib arm [47], thus confirming that RLT outperforms targeted agents in patients progressive on first-line therapy with SSA. Two prospective, randomized, phase II trials (COMPETE and COMPOSE) are currently underway to compare the efficacy of RLT versus everolimus or versus the best standard of care (chemotherapy or everolimus, according to the investigator’s choice) in patients with unresectable progressive GEP-NETs (ClinicalTrials.gov NCT03049189 and NCT04919226).

#### Recommendation

In patients with progressive G1-G2 GEP-NETs, RLT should be preferred as a second-line treatment over targeted agents (everolimus or sunitinib) after the failure of SSA due to its better-expected efficacy and safety profile (2b - B).

#### Q5. What is the evidence for choosing RLT versus chemotherapy after the failure of somatostatin analogs?

Both retrospective and prospective evidences indicate that chemotherapy is effective in treating GEP-NETs [48]. Specifically, alkylating agents such as streptozocin, dacarbazine, and temozolomide (alone or in combination with capecitabine) have demonstrated antitumor activity in panNETs [49–52]. The prospective ECOG-ACRIN E2211 phase II trial recently compared temozolomide alone to temozolomide plus capecitabine in 144 patients with advanced progressive G1-G2 panNETs. The study showed a significant improvement in PFS in the combination arm (median PFS 22.7 vs. 14.4 months respectively) and a trend towards improved ORR (40% vs. 34%) and median OS (58.7 vs. 53.8 months, respectively), although 45% of patients experienced G3/G4 toxicity [53]. While most well-differentiated gastrointestinal NETs tend to be resistant to alkylating agents, fluoropyrimidine-based combinations (e.g., FOLFOX) show antitumor activity in this patient population, potentially causing rapid tumor shrinkage [54–56]. A large, multicenter, retrospective study of 508 patients with advanced GEP-NETs recently showed that second-line therapy with RLT was associated with improved PFS compared to targeted therapies or chemotherapy (median 2.2 years [95% CI, 1.8–2.8 years] vs. 0.6 years [95% CI, 0.4-1.0 years] respectively in the matched population; *P* < 0.001). This effect was consistent across different primary sites and hormonal statuses, though the advantage in PFS was not observed in tumors with a Ki-67 greater than 10% [42]. According to retrospective evidence, RLT is associated with improved survival outcomes in patients who did not receive chemotherapy before RLT initiation [57, 58]. Several clinical trials are currently comparing RLT with chemotherapy in patients with progressive disease (NCT05247905, NCT04919226), and results are eagerly awaited.

Overall, many factors should be considered when choosing between RLT and chemotherapy in patients who are progressive on first-line SSA therapy. These include the pace of tumor growth and the need for rapid tumor shrinkage. While the density of SSTR expression by SSTR-PET scan can accurately preselect the patients most likely to respond to RLT, methylguanine-DNA methyltransferase testing might be helpful in predicting response to temozolomide-based regimens.

#### Recommendation

In patients with progressive G1-G2 GEP-NETs, RLT should be preferred as a second-line treatment over chemotherapy after the failure of SSA. However, chemotherapy remains an option to consider in the treatment of panNET patients who have a high tumor burden and/or the presence of tumor-related symptoms, or in cases of rapid progression, regardless of the primary tumor site (3b - A).

#### Q6. What is the evidence for choosing RLT versus high-dose somatostatin analogs after the failure of standard-dose somatostatin analogs in NF NETs?

While it is well-established that escalating the dose of SSA can enhance symptom control in functioning tumors when the standard SSA dosage proves ineffective, the actual impact of increased SSA dosages on tumor growth, particularly in the clinical context of non-functioning tumors, remains ambiguous. Until recently, selecting a second-line therapy after the standard SSA dose fails in well-differentiated G1-G2 GEP-NETs was notably challenging. Earlier retrospective studies suggested a potential improvement in PFS with increased SSA doses [59]. However, this observation was not corroborated in prospective studies involving patients with radiologically confirmed progressive disease under standard SSA doses. In such clinical scenarios, the reported median PFS values, as indicated by the CLARINET FORTE study [60] and the control arms of the NETTER-1 trial [5], ranged between 5 and 8 months. A recent meta-analysis examining 783 patients in 11 studies found that the proportion of patients experiencing disease progression under high-dose SSA was 62% (with a 95% confidence interval ranging between 53% and 70%) per 100 subjects treated annually [61]. Conversely, in the same clinical scenario of progressive well-differentiated GEP-NETs, RLT demonstrated a significantly higher PFS rate, as observed in both randomized controlled trials and real-world study settings. Data from the phase-3 NETTER-1 trial, where the median PFS was not reached in the initial analysis [5] and was estimated at 25 months in the final analysis [62], aligns with findings from retrospective multicenter studies. These studies reported a median PFS of approximately 2.5 years [12, 42].

A similar trend was observed when considering the ORR as an endpoint. In the context of high-dose SSA, although earlier retrospective small-scale studies reported promising objective response rates of up to 31% [59], prospective trials indicated a significantly lower likelihood of achieving an objective tumor response, with rates ranging between 3 and 4% [5, 60]. On the other hand, when analyzing the ORR for RLT, the values vary significantly. The NETTER-1 study reported a rate of 18% [5], while the larger retrospective study by Brabander et al. indicated a range between 31 and 58% [12].

Based on these considerations, RLT has demonstrated greater efficacy compared to high-dose SSA in the various clinical settings evaluated, including both RCTs and retrospective real-world studies. This superiority is evident in terms of both PFS and ORR.

#### Recommendation

In patients with progressive G1-G2 GEP-NETs, RLT is recommended as a second-line treatment over high-dose SSA after the failure of standard dose SSA due to its better expected efficacy. High-dose SSA remains an option as a temporary bridge until RLT initiation or in patients unfit for other antitumor treatments due to comorbidities (1b - A).

#### Q7. How and when should the efficacy of RLT be monitored after initiating treatment?

3D imaging, particularly through contrast-enhanced CT or MRI, is the main method for evaluating treatment response by observing changes in lesion dimensions over time [18]. Tumor size measurements are primarily conducted according to the Response Evaluation Criteria in Solid Tumours version 1.1 (RECIST 1.1) [63]. However, assessing treatment response based solely on changes in tumor size presents several challenges, especially with GEP-NETs. These tumors may stabilize or initially increase in size even when responding to treatment. Additionally, the occurrence of central tumor necrosis frequently reported during RLT complicates assessments with radiological criteria due to the ‘false-positive’ increases. Furthermore, shrinkage following RLT can be a delayed occurrence [64–66]. These factors underscore the limitations associated with RECIST 1.1 criteria, suggesting that their use in evaluating slow-growing neoplasms such as GEP-NETs should be cautiously approached.

To address these limitations, the Choi criteria have been introduced, assessing both the dimensional changes and the density variation of lesions in CT images with contrast enhancement. Numerous studies comparing the two criteria for NET evaluation consistently show equal or markedly superior results for Choi versus RECIST [67, 68]. However, it is important to note that while the arterial phase of CT is most commonly used in assessing GEP-NETs, considering their vascularity, the Choi criteria rely on images obtained during the portal venous phase [69]. This discrepancy represents a major limitation in applying the Choi criteria in the neuroendocrine context.

In light of these challenges, new methods have been proposed to assess therapy response, including the application of long-established tools used for evaluating growth rates in other neoplastic pathologies [70]. The tumor growth rate (TGR) is one emerging tool based on the variation in the volume of target lesions, normalized for the time between two radiological assessments (CT or MRI). Recent studies have also highlighted its application in the neuroendocrine field [71, 72], showing that baseline TGR highlights the heterogeneity of well-differentiated GEP-NETs and predicts increases in Ki-67 index over time [73].

Additionally, Weber M et al. evaluated the utility of hybrid techniques such as SSTR-PET/MRI in a small sample study. The results suggest that pre-therapeutic SSTR-PET/MRI may not be a reliable predictor of treatment response to RLT in NET patients. Conversely, patients treated with SSA exhibit variations in the apparent diffusion coefficient map on MRI imaging compared to those treated with RLT. Finally, features extracted from SSTR-PET/MRI performed before RLT were not good predictors of treatment response [74].

#### Recommendation

RECIST 1.1 criteria, evaluated by contrast-enhanced CT or MRI, should be used to monitor the efficacy of RLT during follow-up. Attention should also be paid to changes in tumor lesion morphology beyond modifications in their size (3b - A).

#### Q8. How to manage frail patients who have to undergo RLT?

Frailty is a syndrome with complex multifactorial physiopathology affecting up to 17% of the geriatric population [75]. This clinical status implies major vulnerability across multiple health domains, including weakness, decreased functional performance, unintentional weight loss, cognitive impairment, increased risk of comorbidities, and organ dysfunction, leading to adverse health outcomes [75]. As the prevalence of GEP-NETs and the elderly population rate increase globally, it is reasonable to hypothesize that a progressively higher proportion of patients with GEP-NETs will be frail. Data from the Surveillance, Epidemiology, and End Results (SEER) analysis of 29,664 GEP-NET cases showed that the median age at diagnosis was 63 years, with the peak incidence observed at age 80. Additionally, another database analysis of 22,744 cases revealed the highest incidence rate of GEP-NETs in patients over 70 years old, with 16–17 cases per 100,000 [1]. The frail oncological population tends to receive delayed or incomplete diagnostic evaluations and often suboptimal therapy, considering the patient’s comorbidities and major risk of toxicity or complications, leading to an unfavorable therapeutic risk/benefit ratio [76].

Regarding RLT, frail patients more commonly present with altered renal function or hematological disorders, thus tending to be less frequently eligible for RLT. Currently, there are no standardized recommendations in the literature regarding using RLT in frail patients. Theiler et al. conducted a retrospective matched cohort study to assess the efficacy and safety of RLT with 90Y-DOTATOC or 177Lu-DOTATATE in elderly patients over 79 years old affected by well-differentiated G1 or G2, SSTR-positive NETs compared to their younger counterparts. The exclusion criteria included ECOG performance status ≥ 3, hematological impairment (hemoglobin < 80 g/L, platelet count < 75 × 10^9^/L), reduced eGFR (< 45 mL/min), or increased levels of AST/ALT (> 3 times upper range of normal). Overall, despite a higher baseline rate of comorbidities, renal and hematological impairment, and a lower ECOG performance status in the elderly cohort, RLT was found to be an effective strategy with a similar toxicity profile in both groups. Nevertheless, long-term adverse events, particularly renal dysfunction when administered 90Y-DOTATOC rather than 177Lu-DOTATATE, cannot be completely ruled out. No statistically significant differences were observed regarding the OS. The median OS in the elderly and younger group was respectively 3.4 years and 6.0 years (*p* = 0.094) [77]. These results suggest that RLT may be a valid and relatively safe therapeutic option in a carefully selected cohort of frail patients. However, more robust and large-cohort studies are warranted to explore the risk/benefit ratio, also in the long-term, of RLT in this subgroup of patients. Such initiatives would be of remarkable impact, considering that alternative medical options such as targeted drugs (everolimus or sunitinib) or systemic chemotherapy are generally associated with higher toxicity and deterioration of QoL.

An interdisciplinary and multidimensional approach is fundamental to guide therapeutic decisions in such a vulnerable population, especially when standardized guidelines are lacking. To provide the best care for frail individuals, it is necessary to scrupulously identify adequately eligible patients. Therefore, in a multidisciplinary context, validated assessment tools should be implemented to prudently evaluate important domains such as functional, cognitive, and nutritional status, potential limitations in activities of daily living, social settings, and comorbidities.

#### Recommendation

RLT should also be considered in frail patients as a valid therapeutic option despite the lack of specific supporting data. It is reasonable, especially in the elderly population with comorbidities, to pay greater attention to renal function and potential marrow toxicity before initiating therapy (5 - B).

#### Q9. Is there a room for RLT in G3 GEP-NETs?

Retrospective evidence suggested that RLT can be a relevant therapeutic option in patients with SSTR-positive G3 GEP-NETs, leading to disease control rates ranging between 30% and 80% and median PFS between 9 and 23 months [21, 78]. In the recent NETTER-2 trial, which evaluated 226 enrolled patients, 35% had G3 tumors. Overall, treatment with RLT was associated with a significant improvement in PFS (median PFS: 8.5 months in the control arm versus 22.8 months in the investigational arm; stratified HR: 0.28, *p* < 0.0001) and ORR (9.3% in the control arm versus 43% in the investigational arm; stratified OR: 7.81, *p* < 0.0001) [16]. Notably, PFS and ORR improvements were consistent across all pre-specified subgroups, including the G3 subgroup. Based on these results, it is likely that first-line treatment with RLT will be approved soon by regulatory authorities, becoming the first standard treatment option supported by high-level evidence for patients with advanced, G2-G3, SSTR-positive GEP-NETs. Another prospective phase III trial, the COMPOSE trial, is currently underway to compare first or second-line RLT versus the best standard of care (chemotherapy or everolimus according to investigator’s choice) in patients with either G2 or G3 unresectable SSTR-positive GEP-NETs [79]. The trial results are eagerly awaited, as they will provide much-needed information on treatment sequencing also in patients with G3 GEP-NETs.

No high-level evidence of antitumor activity currently exists for treatment modalities alternative to RLT in patients with metastatic G3 GEP-NETs. According to retrospective data [80] and in light of the recent results of the NETTER-2 trial [16], SSA may exert some antiproliferative activity in patients with G3 GEP-NETs, although with significantly inferior outcomes compared to RLT. On the other hand, small series have documented the activity of either sunitinib [81] or everolimus (alone or in combination with temozolomide) in G3 GEP-NETs [82]. Alkylating-based (i.e., CAPTEM or STZ/5-FU) and fluoropyrimidine-based (i.e., FOLFOX) chemotherapy protocols appear effective in patients with G3 GEP-NETs [83]. According to retrospective evidence, the CAPTEM regimen is associated with a median PFS ranging between 9 and 15 months in patients with advanced G3 tumors of the digestive tract [84, 85]. Responses to temozolomide-based regimens appear more frequent in the first-line setting and in pancreatic primaries. The efficacy of etoposide-platinum chemotherapy appears limited in advanced G3 NETs, with the response rate in this population inferior to that observed in patients with poorly differentiated NECs [86].

Overall, RLT might be currently considered as a preferred option in the first-line treatment of patients with advanced SSTR-positive G3 GEP-NETs. Chemotherapy, particularly alkylating-based regimens, might be reserved to SSTR-negative G3 NETs or to patients progressing on RLT.

#### Recommendation

As soon as RLT is approved by regulatory authorities, it should be considered a valid option for patients with G2-G3 GEP-NETs expressing SSTR (1b - A).

#### Q10. Is there a rationale for repeating RLT treatment?

The rationale for repeating RLT in patients with GEP-NETs involves several factors. The decision is typically individualized, based on a combination of clinical assessments, imaging, and biochemical evaluations.

If there is evidence of disease progression or recurrence following the initial course of RLT, a repeat treatment may be considered to target new or recurrent lesions. Initially, an SSTR-PET evaluation should be conducted to confirm the presence of somatostatin receptors on the NET lesions. According to the Delphi consensus, a partial response or stable disease must have been achieved for at least one year after the first RLT treatment [13]. To accurately determine which patients could benefit from retreatment, implementing dosimetry in clinical practice is crucial. Dosimetry correlates tumor-absorbed doses and treatment effectiveness, especially in larger tumors [87, 88].

Recent studies have demonstrated the safety and efficacy of an RLT rechallenge with dosimetry calculations based on healthy organs such as the kidneys and bone marrow [89–91]. These findings suggest that incorporating personalized dosimetry, aimed at identifying organs with dose limits and determining the maximum tolerated accumulated activity, can enhance standard clinical practices by ensuring that therapeutic doses stay within safe limits for healthy organs. Notably, patients who reached the maximum tolerable absorbed dose of 23 Gy in their kidneys experienced nearly double the median PFS and OS [92]. This highlights the significant potential benefits of adopting a personalized approach over fixed dosing in terms of oncological outcomes.

The decision to repeat RLT is complex and requires careful consideration of various factors. Regular follow-up assessments, imaging studies, and ongoing communication between the patient and the dedicated tumor board are crucial for determining the most appropriate course of action in managing NETs.

#### Recommendation

Although not yet approved by regulatory authorities, retreatment with RLT should be considered a valid therapeutic option for those patients who had a favorable response to initial RLT at the time of disease progression. Dosimetry data, including initial RLT, should be used to tailor the personalized dose for the retreatment approach (3b - B).

## Conclusions

This position paper strongly advocates for the early integration of RLT into the treatment regimen for advanced SSTR-positive GEP-NETs following the failure of SSA. Before initiating RLT, [18 F]FDG PET/CT is recommended for patients with heterogeneous uptake on SSTR-PET or those suspected of rapid tumor progression. RLT with 177Lu-DOTATATE stands out as the preferred second-line treatment over targeted therapies, chemotherapy, or high-dose SSA for progressive G1-G2 GEP-NETs thanks to its superior efficacy and safety profile. This recommendation applies provided that the disease homogeneously expresses SSTRs, is not rapidly progressing, or is not highly symptomatic. To assess the effectiveness of RLT, RECIST 1.1 criteria through contrast-enhanced CT or MRI are advised, emphasizing changes in tumor morphology. Looking forward, it is anticipated that upon regulatory approval, RLT will be considered a valid treatment option for patients with well-differentiated high-grade SSTR-positive GEP-NETs. Additionally, retreatment with RLT will be suggested for those who have shown a favorable response to the initial treatment upon disease progression, ideally using tailored dosimetry. The key messages from this position paper are summarized in Table 2.

**Table 2:** Key messages and future perspective

- Early RLT should be considered for GEP-NET patients expressing SSTRs who show progression despite SSA therapy.
- In cases of G1-G2 GEP-NETs expressing SSTRs without rapid progression, RLT proves superior to other therapeutic approaches and should be the preferred option upon progression following the initial treatment strategy.
- Alternative therapeutic options, such as chemotherapy (CAPTEM or 5FU-STZ regimens) or targeted therapies, should be considered for rapidly progressing tumors or when there is a significant symptomatic tumor burden, especially in pancreatic NETs.
- It is anticipated that in the near future, RLT will also become available for G3 tumors, as well as a potential retreatment option for patients who responded favorably to initial RLT.
- A multidisciplinary approach to managing GEP-NET patients is strongly recommended, also to identify the most suitable candidates for RLT.

## Electronic supplementary material

Below is the link to the electronic supplementary material.

Supplementary Material 1
