# Supplementary material for: Radioligand therapy in the therapeutic strategy for patients with gastro-entero-pancreatic neuroendocrine tumors: a consensus statement from the Italian Association for Neuroendocrine Tumors (Itanet), Italian Association of Nuclear Medicine (AIMN), Italian Society of Endocrinology (SIE), Italian Association of Medical Oncology (AIOM)
Source: J Endocrinol Invest. 2024 Oct 12;48(1):23–36. doi: 10.1007/s40618-024-02448-6 (PMC11729074; doi:10.1007/s40618-024-02448-6)
Supplement: Supplementary file 1 — Supplementary Material 1 [file 40618_2024_2448_MOESM1_ESM.docx]

**Supplementary Table 1. Level of evidence for therapy and diagnosis.**

| **Grade** | **Therapy** | **Diagnosis** |
| --- | --- | --- |
| 1a | Systematic Review (with homogeneity) of RCT | Systematic review (SR) (with homogeneity) of Level 1 diagnostic studies; Clinical Decision Rules/ Validating cohort study with good reference standards |
| 1b | RCT (with narrow confidence intervals) |  |
| 2a | Systematic review of cohort studies | SR (with homogeneity) of Level >2 diagnostic studies |
| 2b | Individual cohort studies or low quality RCT | Exploratory cohort study with good reference standards; Clinical Decision Rule (CDR) after derivation. |
| 3a | Systematic review (with homogeneity) of case-controlled studies | SR (with homogeneity) of 3b and better studies |
| 3b | Individual case-controlled studies | Non-consecutive study; or without consistently applied reference standards |
| 4 | Case series (and poor quality case-controlled studies) | Case-control study, poor or non-independent reference standard |
| 5 | Expert opinion with no explicit critical appraisal. | Expert opinion without explicit critical appraisal. |

Grade of recommendation: A =Strong; B =Moderate, C =Low, D =Very low
